# Construction of Inverse Metal–Zeolite Interfaces via Area-Selective Atomic Layer Deposition

**DOI:** 10.1021/acsami.1c15569

**Published:** 2021-10-20

**Authors:** Peng Zhai, Laibao Zhang, David A. Cullen, Divakar R. Aireddy, Kunlun Ding

**Affiliations:** † Department of Chemical Engineering, Louisiana State University, Baton Rouge, Louisiana 70803, United States; ‡ Center for Nanophase Materials Sciences, Oak Ridge National Laboratory, Oak Ridge, Tennessee 37831, United States

**Keywords:** zeolite, metal nanoparticles, confinement, inverse catalyst, atomic layer deposition

## Abstract

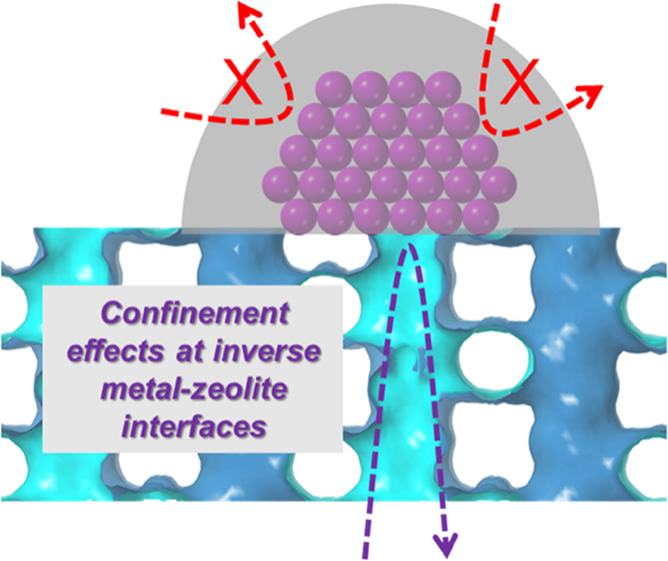

The spatial confinement at metal–zeolite interfaces offers a powerful knob to steer the selectivity of chemical reactions on metal catalysts. However, encapsulating metal catalysts into small-pore zeolites remains a challenging task. Here, we demonstrate an inverse design of metal–zeolite interfaces, “*metal-on-zeolite,*” constructed by area-selective atomic layer deposition. This inverse design bypasses the intrinsic synthetic issues associated with metal encapsulation, offering a potential solution for the fabrication of task-specific metal–zeolite interfaces for desired catalytic applications. Infrared spectroscopy and several probe reactions confirmed the spatial confinement effects at the inverse metal–zeolite interfaces.

## Introduction

Encapsulating metal catalysts inside crystalline microporous materials such as zeolites and metal-organic frameworks not only significantly enhances the thermal stability of metal species but also endows the active sites with unique size/shape/chemoselectivity in heterogeneous catalysis, thanks to the confinement effects at metal-support interfaces.^1−18^ Conventional approaches exclusively adopt “*metal-in-zeolite*” configurations to achieve metal–zeolite interfaces, including immobilizing metal precursors in presynthesized zeolites by impregnation, ion exchange, grafting, and deposition;^3−8^ incorporating metal precursors during zeolite synthesis;^10,19,20^ epitaxial growth of zeolite shell on zeolite-supported metal nanoparticles (NPs);^21,22^ and solid-state crystallization or phase transformation of encapsulating matrices^9,23−25^ These approaches are associated with many intrinsic synthetic issues. For instance, the size mismatch between many metal precursors and zeolite apertures has greatly hindered the encapsulation of metal species into many small-pore zeolites for potential catalytic applications.^19,20^ Moreover, the presence of external anchoring sites on zeolites may lead to incomplete encapsulation of metal species, which compromises the catalytic performance. Organic modifiers, or in other words, poisons, were employed to selectively block nonencapsulated metal surfaces to enhance catalytic selectivity.^26,27^ However, the organic modifiers cannot survive under harsh conditions such as high-temperature calcination/reduction. The poor stability of metal precursors or NPs under harsh hydrothermal conditions for zeolite synthesis represents a grand challenge for the latter approaches involving zeolite growth on metal species.^19,20^ Therefore, the success of these approaches is limited to certain types of metals and zeolites. To construct metal–zeolite interfaces with a broader range of metals and zeolites, a metal- and zeolite-independent synthesis approach is highly desirable.

**Figure 1:**
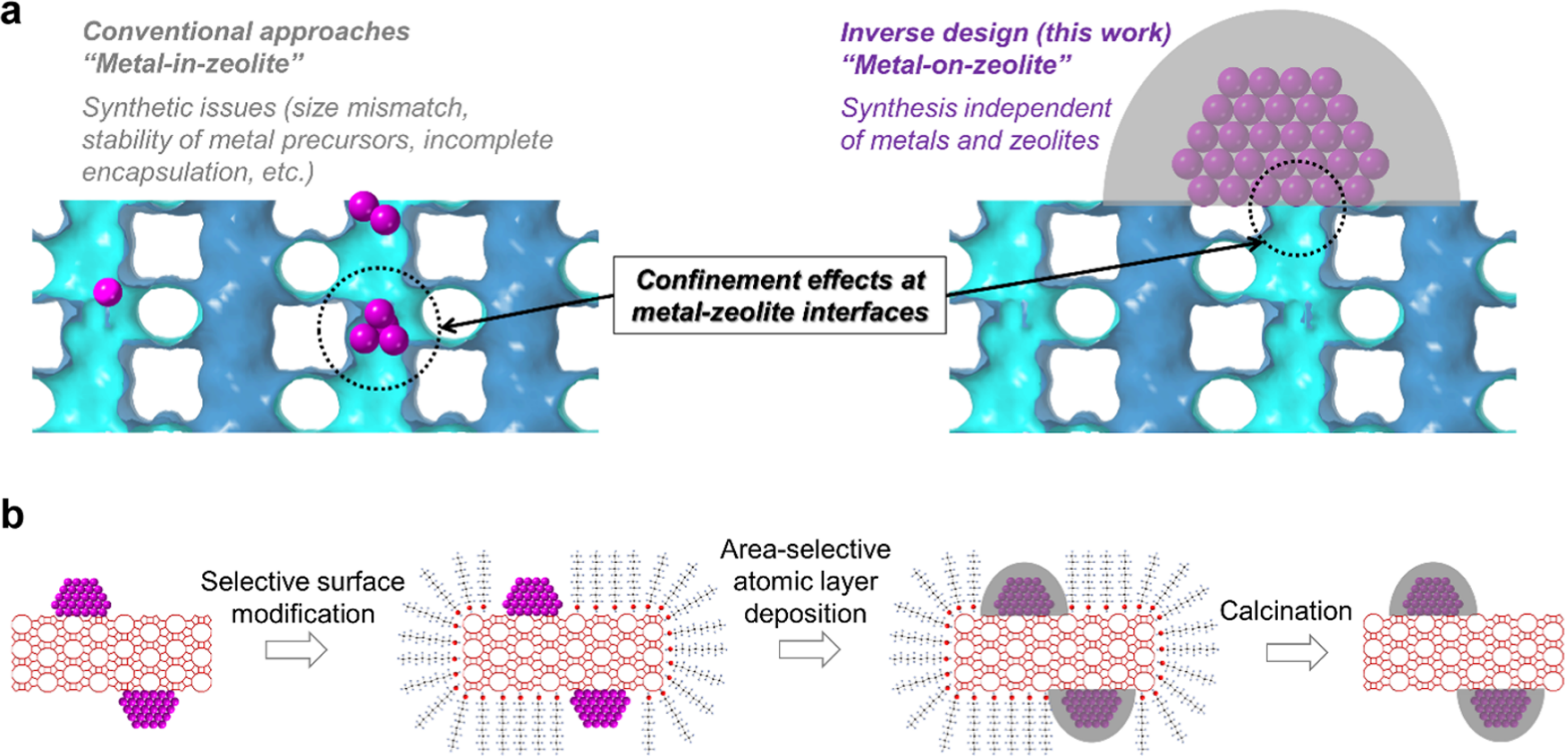
Schematic illustration of the inverse design of metal–zeolite interfaces and synthesis strategy. (a) Illustration of the inverse design and comparison with conventional approaches. (b) Illustration of synthesis steps of inverse metal–zeolite interfaces.

In principle, the aforementioned limitations associated with “*metal-in-zeolite*” configurations can be overcome by an inverse design of metal–zeolite interfaces with a “*metal-on-zeolite*” configuration (Figure 1a). Different from most metal-support interfaces, which are inaccessible to reactants because of the nonporous nature of metal NPs and supports, when metal NPs are deposited on the external surfaces of zeolites, the interface between metal NPs and zeolites are accessible via zeolite pores. By selectively blocking the noninterfacial metal surfaces, the only path that remains accessible to the metal catalysts is through zeolite pores, resulting in spatial confinement effects in catalysis, *i.e*., only the reactants that are smaller than the pore sizes of zeolites can access the metal surfaces and undergo catalytic conversions. This inverse design bypasses the synthetic issues associated with metal encapsulation, especially in small-pore zeolites. Moreover, the inverse design is independent of the types of metals or zeolites, thus offering a robust synthesis protocol to construct task-specific metal–zeolite interfaces for target applications, which is beyond the capability of conventional “*metal-in-zeolite*” approaches.

Here, we report a general design and fabrication of inverse metal–zeolite interfaces for catalytic applications. By manipulating the site selectivity of surface modification and atomic layer deposition, we were able to selectively block the noninterfacial metal sites while leaving the metal–zeolite interfaces accessible for catalysis. The confinement effects at the metal–zeolite interfaces were verified in several probe reactions.

## Results and Discussion

Our synthesis protocol is illustrated in Figure 1b. Metal NPs were first deposited onto the external surfaces of zeolite crystals (synthesis details provided in the Experimental Section); then the zeolite’s external surfaces were selectively modified by organic blocking agents to prevent the deposition of metal oxides onto the zeolite surfaces in the subsequent step; a dense metal oxide shell was then selectively deposited onto metal NPs to block the noninterfacial metal sites; after removing the organic blocking agents by calcination, zeolite surfaces were opened so that reactants were able to access metal–zeolite interfaces through zeolite pores.

Six types of supports were selected to validate the proposed concept, including a nonporous nanospherical alumina and five zeolites with different aperture sizes: SAPO-34 (CHA structure) as an 8-membered ring (MR) zeolite; ZSM-5 (MFI structure), ZSM-35 (FER structure), and ZSM-22 (TON structure) as 10-MR zeolites; and β (BEA structure) as a 12-MR zeolite. The scanning electron microscopy (SEM) images and nitrogen sorption results are provided in Figures S1 and S2, and Table S1.

**Figure 2:**
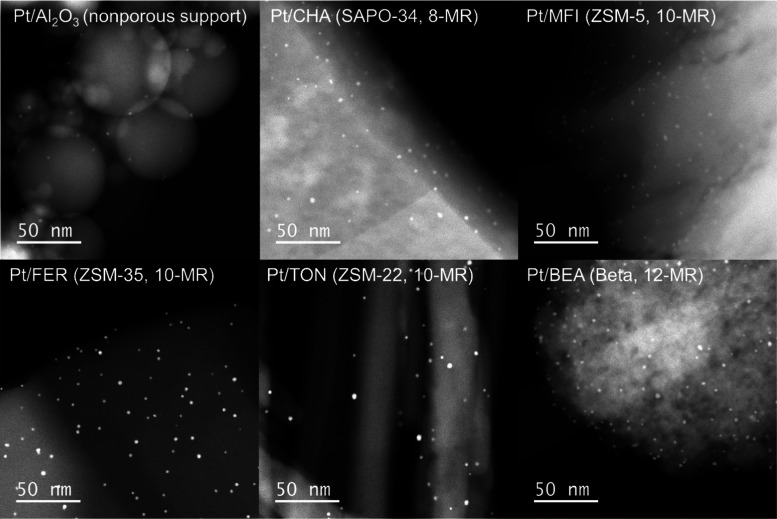
Electron microscopy analysis of the supported Pt NPs. HAADF-STEM images of Pt NPs supported on Al_2_O_3_ and five zeolites.

Colloidal Pt NPs with a size of ∼3 nm were synthesized with the assistance of polyethylenimine and adsorbed onto these supports following our previous work.^28^ Polyethylenimine was removed by calcination in air before carrying out selective surface modification. High-angle annular dark-field scanning transmission electron microscopy (HAADF-STEM) images of the six supported Pt catalysts show monodispersed Pt NPs uniformly distributed on the surfaces of these supports (Figures 2 and S3–S8).

The biggest challenge to construct the target inverse metal–zeolite interfaces is selectively blocking the noninterfacial metal sites while leaving the metal–zeolite interfaces accessible. In principle, this can be achieved by carrying out area-selective atomic layer deposition (AS-ALD) to create a dense layer of metal oxide on the surface of metal NPs rather than zeolites. In a typical AS-ALD synthesis, a blocking agent is needed to modify the substrate surfaces to prevent deposition in the blocked area^29−34^ Long-chain chlorosilane (dodecyldimethylchlorosilane) was chosen as a blocking agent because it selectively bonds to oxide surfaces via a reaction with hydroxyls. The infrared (IR) spectrum of the silane-modified Pt/BEA shows strong C–H bands between 2800 and 3000 cm^–1^, indicating the successful grafting of silane molecules (Figure S9). Meanwhile, the appearance of a negative IR band at 3735 cm^–1^ confirms the consumption of hydroxyls upon silane grafting. IR spectroscopy was further used to study the adsorption of CO molecule to probe the accessibility of Pt surface. No noticeable decrease in CO band intensity was observed after silane grafting (Figure S10), suggesting that the silane groups were mostly grafted on zeolite rather than Pt surfaces.

**Figure 3:**
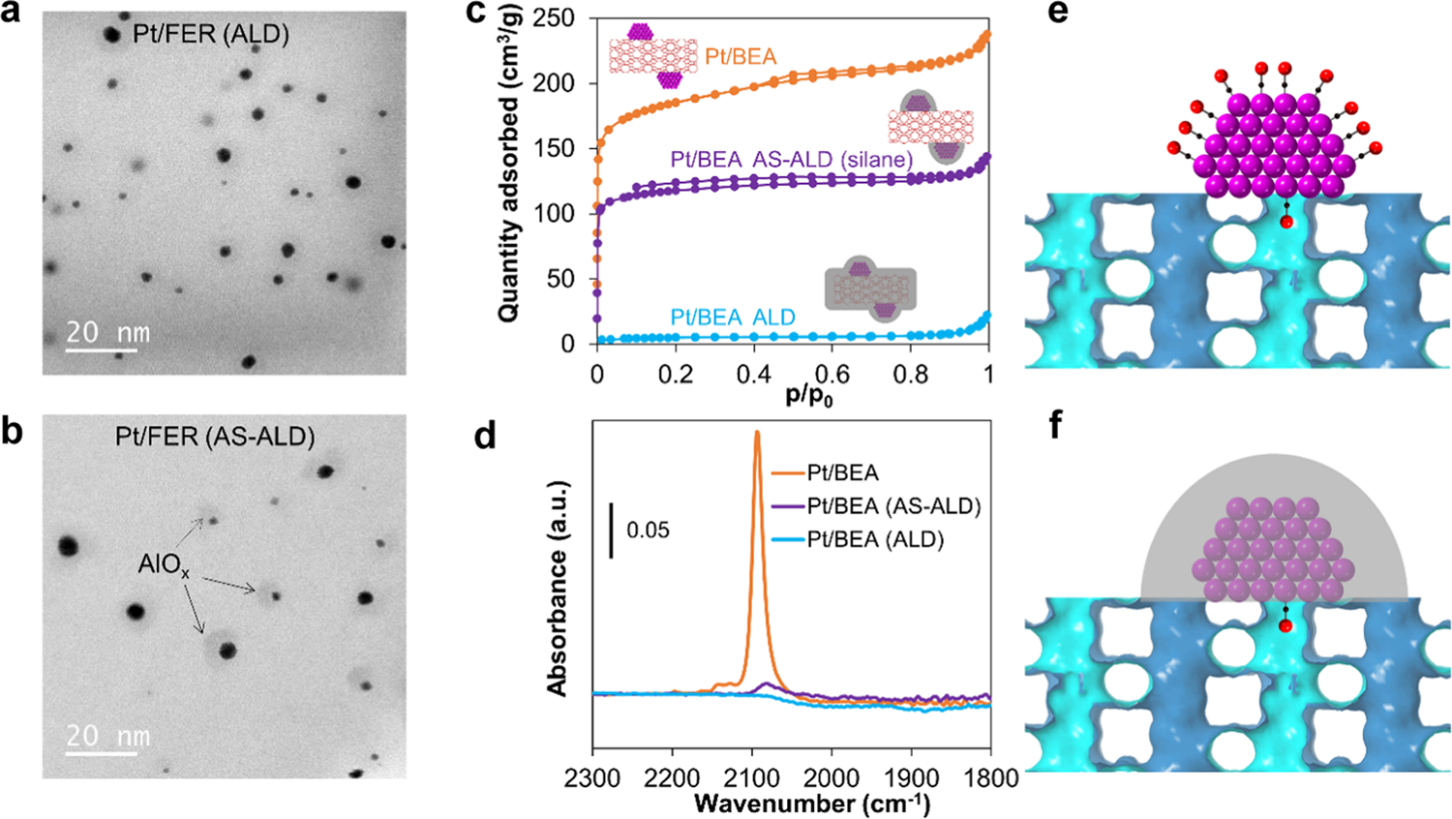
Electron microscopy, nitrogen sorption, and IR studies of the Pt/zeolite after ALD and AS-ALD. (a) and (b) Bright-field STEM images of Pt/FER after ALD and AS-ALD. (c) Nitrogen sorption isotherms and schematic illustrations of uncoated Pt/BEA and that after ALD and AS-ALD. (d) IR spectra of CO molecules adsorbed on uncoated Pt/BEA and that after ALD and AS-ALD. (e) and (f) Illustration of the confinement effect on CO adsorption at Pt-zeolite interfaces.

Then, 20-cycle AlO*_x_* ALD was carried out to encapsulate the supported Pt NPs. The area-selective deposition of AlO_x_ on Pt rather than zeolite surfaces was confirmed by electron microscopy, nitrogen sorption, and IR studies. Pt/FER was chosen for electron microscopy studies because the uniform contrast of FER zeolite with 2D morphology allows one to distinguish the thin shell of oxide overcoat on Pt NPs. As shown in Figures 3a and S11, Pt/FER without silane modification shows even contrast after ALD, with barely noticeable halo structures around Pt NPs, implying that AlO*_x_* ALD is nonselective on Pt or zeolite surfaces without silane groups. In contrast, clear halo structures with a thickness of 3 ± 1 nm were observed around Pt NPs on FER zeolite after AS-ALD (with silane modification, as shown in Figures 3b and S12), which confirms that the presence of silane groups on zeolite surfaces is able to selectively inhibit the growth of AlO*_x_* on zeolite rather than Pt surfaces.

AlO*_x_* ALD overcoat is known to generate micropores upon annealing at 600 °C or above,^35,36^ which is undesirable for our design. Therefore, the calcination temperature was kept at 500 °C in this work to avoid potential cracking of ALD overcoat. The complete blockage of zeolite pores by 20-cycle AlO*_x_* ALD without silane modification was verified by nitrogen sorption, evidenced by the dramatic reduction in specific surface areas and pore volumes (Figures 3c, S2, and Table S1). For instance, the specific surface area and pore volume of Pt/BEA reduced from 546 m^2^/g and 0.36 cm^3^/g to 16 m^2^/g and 0.03 cm^3^/g, respectively. With silane modification, the specific surface area and pore volume of Pt/BEA were largely preserved (340 m^2^/g and 0.22 cm^3^/g) upon AS-ALD and silane removal, indicating that the internal surface of BEA zeolite remained mostly accessible. The decrease in zeolite surface area is likely originated from the deposition of AlO*_x_* on zeolite surfaces, which partially blocks access to zeolite pores. The area selectivity of AlO*_x_* ALD may be further improved by adopting different AlO*_x_* precursors^37^ or optimizing ALD parameters^38^ thus preserving the access to more zeolite pores.

The accessibility of Pt-zeolite interfaces through zeolite pores was verified by IR spectroscopy with CO as a probe molecule. As shown in Figure 3d, the IR band intensity associated with linear CO on Pt surface decreased by ∼95% after AS-ALD and silane removal. In contrast, the CO band intensity completely diminished after ALD without silane modification. The diameter of the biggest aperture (12-MR) in BEA zeolite is 6 Å, which allows only a few Pt atoms to be exposed, meaning that each zeolite aperture is able to accommodate one or a few CO molecules to be adsorbed at the Pt-zeolite interface (Figure 3e,f). This explains the dramatic decrease in CO band intensity. On the other hand, the CO band center shifted from 2093 to 2081 cm^–1^ after AS-ALD and silane removal, which is in concert with the decrease of dipole–dipole coupling between adjacent CO molecules because of the interruption of CO patch formation on Pt surface. The partial blockage of zeolite pores because of the imperfect AS-ALD might also contribute to the decrease in CO IR intensity.

In addition to chlorosilane, which modifies the zeolite surface via covalent bond formation with surface hydroxyls, another type of blocking agent, 1-hexadecanol, was also used for AS-ALD in this work. 1-hexadecanol was selectively adsorbed onto zeolite surfaces from a pentane solution via hydrogen bonding with surface hydroxyl, as evidenced by IR spectroscopy (Figure S13). The electron microscopy (Figure S14), nitrogen sorption (Table S1 and Figure S15), and CO IR (Figures S16 and S17) results are similar to that of chlorosilane-modified Pt/zeolite, indicating the successful selective deposition of AlO*_x_* on Pt surfaces via AS-ALD, while the Pt-zeolite interfaces remained accessible through zeolite pores. It is worth noting that the preserved accessible surface area and pore volume after AS-ALD with 1-hexadecanol as a blocking agent are slightly smaller than that of chlorosilane-assisted AS-ALD. This implies that the area selectivity of AlO*_x_* ALD in the presence of a hydrogen-bonded blocking agent is lower than the covalent-bonded chlorosilanes.

**Figure 4:**
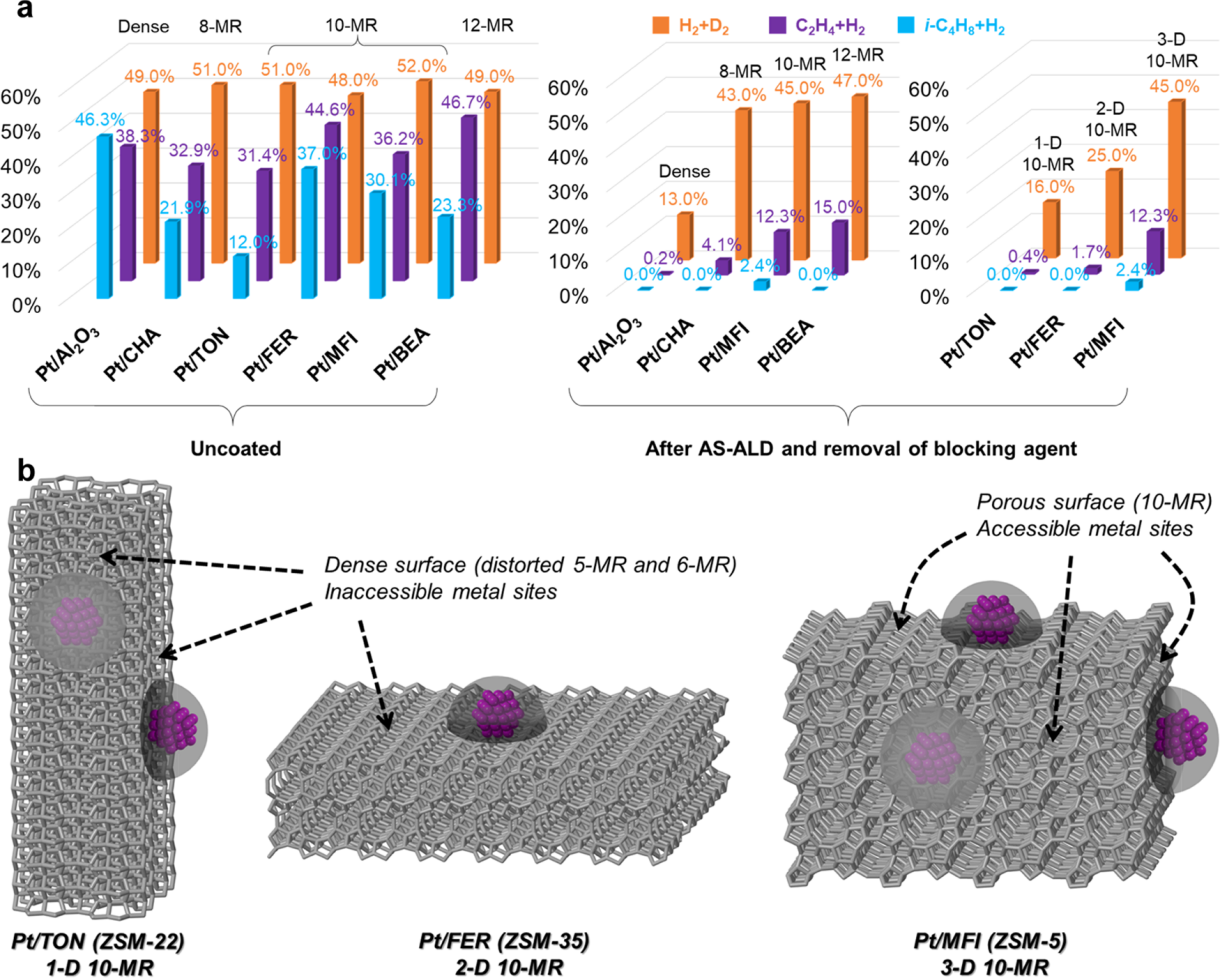
Catalysis studies and illustration of zeolite structures. (a) Catalytic performance of supported Pt NPs in H/D exchange between H_2_/D_2_ and hydrogenation of ethylene and isobutene before and after AS-ALD. (b) Structural origin of the difference in catalytic activity of Pt/TON, Pt/FER, and Pt/MFI after AS-ALD.

Three catalytic reactions were investigated to probe the confinement effect at Pt-zeolite interfaces, including H/D exchange between H_2_ and D_2_, ethylene (C_2_H_4_) hydrogenation, and isobutene (*i*-C_4_H_8_) hydrogenation. These reactions were selected based on the kinetic diameters of reactant molecules (2.9, 3.9, and 4.8 Å for H_2_/D_2_, C_2_H_4_, and *i*-C_4_H_8_, respectively).^39,40^ By comparing the catalytic performance of Pt/Al_2_O_3_ after different numbers of ALD cycles (Figure S18), we determined that 20 ALD cycles were necessary to completely encapsulate Pt NPs. The C_2_H_4_ and *i*-C_4_H_8_ conversions on Pt/Al_2_O_3_ dropped from 38 and 46% to 0.3% and 0, respectively, after 20-cycle AlO*_x_* ALD. Because of the dense nature of the Al_2_O_3_ support, Pt/Al_2_O_3_ also exhibited negligible catalytic activity after AS-ALD and removal of blocking agents (Table 1 and Figure 4a), identical with that after ALD.

**Table 1:** Catalytic Performance of Supported Pt NPs in H/D Exchange Between H_2_/D_2_ and Hydrogenation of Ethylene and Isobutene

	uncoated			after AlO*_x_* ALD			after AlO*_x_* AS-ALD (chlorosilane)		
	*i*C_4_H_8_ + H_2_	C_2_H_4_ + H_2_	H_2_/D_2_	*i*C_4_H_8_ + H_2_	C_2_H_4_ + H_2_	H_2_/D_2_	*i*C_4_H_8_ + H_2_	C_2_H_4_ + H_2_	H_2_/D_2_
Pt/Al_2_O_3_	46.3	38.3	49.0	0.0	0.3	8.0	0.0	0.2	13.0
Pt/CHA	21.9	32.9	51.0	0.0	0.3	10.0	0.0	4.1	43.0
Pt/TON	12.0	31.4	51.0	0.0	0.3	2.0	0.0	0.4	16.0
Pt/FER	37.0	44.6	48.0	0.0	0.3	7.0	0.0	1.7	25.0
Pt/MFI	30.1	36.2	52.0	0.0	0.8	3.0	2.4	12.3	45.0
Pt/BEA	23.3	46.7	49.0	0.0	1.9	19.0	0.0	15.0	47.0

Moving from the dense Al_2_O_3_ support to zeolite supports with 8-MR (CHA, 3.8 Å), 10-MR (MFI, 4.7 Å), and 12-MR (BEA, 6 Å) apertures, the conversion of H_2_/D_2_ exchange sharply increased to nearly equilibrium conversion (∼50%) after AS-ALD and silane removal, as H_2_ and D_2_ molecules can easily enter zeolite pores bigger than 6-MR. Meanwhile, the C_2_H_4_ conversion gradually increased to 4.1, 12, and 15%, respectively, which is also expected since the kinetic diameter of C_2_H_4_ molecule is comparable with 8-MR but smaller than 10-MR and 12-MR. In contrast, the Pt/zeolite catalysts after ALD without using surface blocking agents show negligible activity, similar to that of ALD-coated Pt/Al_2_O_3_ (Figure S19). These trends in H_2_/D_2_ exchange and C_2_H_4_ hydrogenation activity agree well with the kinetic diameters of reactant molecules and zeolite aperture sizes. To our surprise, these Pt/zeolite catalysts, including Pt/BEA, whose aperture size is greater than the kinetic diameter of *i*-C_4_H_8_ (6 Å vs 4.8 Å), remained mostly inactive in *i*-C_4_H_8_ hydrogenation. Similar trends were observed when 1-hexadecanol was employed as a blocking agent for AS-ALD (Figure S20). One possible explanation is that the AlO*_x_* ALD precursor, trimethylaluminum (5.5–6 Å), is able to enter the 12-MR aperture of BEA zeolite and cause shrinkage in pore size, which limits the access of *i*-C_4_H_8_ molecule.

We next compare the catalytic performance of three 10-MR zeolites with 3-dimensional (MFI), 2-dimensional (FER), and 1-dimensional (TON) channels. Zeolites tend to adopt morphologies with large dense surfaces to minimize their surface energies. For instance, TON zeolite usually adopts 1-dimensional rod or wire morphology (SEM image in Figure S1) with the largest sides composed of 5-MR and distorted 6-MR (Figure 4b), and molecules can only enter TON zeolite from the two ends of the 1-dimensional structure. Similarly, a typical morphology of FER zeolite is a 2-dimensional sheet or plate (SEM image in Figure S1), whose largest facets are composed of 5-MR (Figure 4b), meaning that molecules can only access the internal surface of FER zeolite from the thin lateral surface of the 2-dimensional crystals. Since Pt NPs mostly sit on the largest surfaces of the TON and FER zeolites (Figures 2, S5, and S6), these Pt-zeolite interfaces are inaccessible to reactant molecules. Therefore, Pt/TON and Pt/FER showed very low activity in C_2_H_4_ and *i*-C_4_H_8_ hydrogenations after AS-ALD and blocking agent removal (Table 1, Figures 4a and S20). The slightly higher activity of Pt/FER in C_2_H_4_ hydrogenation might be originated from the small number of Pt NPs sitting on the thin lateral surfaces of FER sheets/plates with 10-MR or 8-MR apertures (Figures 2 and S6). For the same reason, Pt/FER also exhibited slightly greater activity in H_2_/D_2_ exchange compared to Pt/TON after AS-ALD and blocking agent removals. The latter basically behaved like Pt/Al_2_O_3_. Such crystal morphological effect in catalysis cannot be observed on conventional “*metal-in-zeolite*” configurations because reactant molecules can access the encapsulated metal sites through the largest apertures.

## Conclusions

In conclusion, we have presented an inverse design of metal–zeolite interfaces with a “*metal-on-zeolite*” configuration, which can overcome the intrinsic synthetic issues associated with “*metal-in-zeolite*” configurations. This inverse design of metal–zeolite interfaces is, in principle, independent of the types of metals and zeolites. It could potentially enable the construction of task-specific metal–zeolite interfaces for desired catalytic applications. Furthermore, since the metal NPs sit on the external surfaces of zeolite crystals, and the aperture size is dependent on the crystal facets of zeolites, unusual crystal morphological effects were observed on low-dimensional zeolites. This inverse design of metal–zeolite interfaces may also be extended to other types of microporous materials such as metal-organic frameworks, providing a powerful tool to tailor the confinement effects in heterogeneous catalysis. Moreover, this inverse design may find applications in zeolite-based gas sensors^41^ by discriminating the access of gas molecules with different sizes.

## Experimental Section

### Materials

Al_2_O_3_ (NanoDur, 30–40 m^2^/g) was purchased from Alfa Aesar; Zeolites β (CP811C-300) and ZSM-5 (CBV28014) were obtained from Zeolyst International; ZSM-22 and ZSM-35 were ordered from ACS Material. K_2_PtCl_4_ (99.9%) was purchased from Strem Chemicals, Inc. Acetone (99.5%) was purchased from Millipore Corporation. Phosphoric acid (H_3_PO_4_, 85%) was purchased from VWR. Polyethylenimine (PEI, *M*_w_ = 25000 by LS, *M*_n_ =10000 by GPC), NaBH_4_ (99%), pentane (anhydrous, 99%), dodecyldimethylchlorosilane (95%), 1-hexadecanol (99%), trimethylaluminum (TMA, 97%), tetraethylammonium hydroxide solution (TEAOH, 40 wt % in H_2_O), aluminum-tri-sec-butoxide (97%), and LUDOX HS-30 colloidal silica (30 wt % suspension in H_2_O) were purchased from Sigma-Aldrich. Deionized water obtained from an EMD Millipore Milli-DI Water Purification System was used in all experiments.

Ethylene (5% in N_2_, UHP), isobutene (5% in N_2_, UHP), hydrogen (5% in N_2_, UHP), deuterium (5% in N_2_, UHP), nitrogen (UHP), hydrogen (UHP), argon (UHP), helium (UHP), CO (5% in helium, UHP), oxygen (10% in argon, UHP), air (ultra zero) and N_2_ (research plus) were provided by Airgas company.

### Synthesis of Zeolite SAPO-34 (CHA)

The synthesis of SAPO-34 was adapted from the literature:^42^ 8.57 g of aluminum-tri-sec-butoxide was dissolved in a mixture of 22.52 g of TEAOH and 31.3 g of H_2_O. Then 1.99 g of colloidal silica (30 wt %) was added to the mixture under 600 rpm stirring to get a clear solution. Subsequently, 8.19 g of H_3_PO_4_ was added dropwise and immediately formed a white colloidal dispersion. The molar ratio of Al_2_O_3_/P_2_O_5/_SiO_2_/TEAOH was 1:2:0.6:4. The colloidal dispersion was transferred into a 100 mL Teflon-lined autoclave and heated at 180 °C for 24 h under stirring (300 rpm). The solid product was centrifuged and washed with distilled water at least five times and dried at 60 °C for 12 h. The product was calcined in air at 600 °C for 5 h (ramping rate of 1.5 °C/min) to remove the organic species.

### Synthesis of Colloidal Pt NPs

The synthesis of colloidal Pt NPs was adapted from our previous work^28^ Briefly, 10.3 g of 5 mM K_2_PtCl_4_ aqueous solution was added to 3 wt % PEI aqueous solution under stirring (PEI/Pt = 0.05, mol/mol). The obtained solution was sonicated for 5 min and then left at room temperature under stirring for 12 h for complexation. The colorless solution was bubbled by N_2_ 10 min to remove dissolved oxygen; 4 wt % NaBH_4_ aqueous solution (NaBH_4_/Pt = 10) was then added under stirring. After 1 h of reduction, a dark-brown colloidal dispersion was obtained.

### Adsorption of Colloidal Pt NPs

Colloidal Pt NPs were adsorbed onto Al_2_O_3_ and zeolites via direct adsorption or antisolvent-induced adsorption.^28^ For the direct adsorption of PEI-Pt NPs on Al_2_O_3_, zeolite β, SAPO-34, ZSM-22, and ZSM-35, 1.5 g of support was dispersed in 3 mL of water, then the as-synthesized PEI-Pt colloidal dispersion was added and vigorously mixed for 2 min (Pt loading of 0.25 wt % for all of the samples). The obtained mixture was stirred for 10 min and centrifuged, washed with water three times and twice with pure acetone, and dried at room temperature overnight.

For the antisolvent-induced adsorption of PEI-Pt NPs onto ZSM-5, 1.5 g of ZSM-5 was dispersed in 3 mL of water; then, the as-synthesized PEI-Pt colloidal dispersion was added and vigorously mixed for 2 min (Pt loading of 0.25 wt %). Briefly, 7.4 mL of acetone (acetone/H_2_O = 1:1, v/v) was added under stirring to induce the complete adsorption of Pt NPs onto ZSM-5. The obtained suspension was stirred for 10 min and centrifuged, washed with water three times and twice with pure acetone, and dried at room temperature overnight.

All of the supported Pt NPs were calcined in air at 400 °C for 4 h with a ramping rate of 1.5 °C/min.

### Surface Modification

The surface density of dodecyldimethylchlorosilane and 1-hexadecanol for surface modification was 0.75 and 1.5/nm^2^, respectively. Calculations were based on the external surface area of supports measured by nitrogen sorption.

In a typical synthesis, 100 mg of Pt/β (external surface area of 185 m^2^/g) was dried at 150 °C overnight and dispersed in 4.52 mL of anhydrous pentane in a glass vial, sonicated for 2 min, and stirred for 10 min. Then 0.48 mL of dodecyldimethylchlorosilane/pentane solution (0.05 mM/mL) was added while stirring. After stirring for 10 min, the solid was separated by centrifugation, washed with pentane three times, and then dried at room temperature.

For 1-hexadecanol modification, 100 mg of Pt/β was dried at 150 °C overnight and dispersed in 3.15 mL of anhydrous pentane in a glass vial, sonicated for 2 min, and stirred for 10 min. Then 1.85 mL of 1-hexadecanol/pentane solution (0.01 g/g) was added while stirring. After stirring for 10 min, the solid was separated by centrifugation, washed with pentane three times, and then dried at room temperature.

### Atomic Layer Deposition

Al_2_O_3_ ALD was conducted on a benchtop ALD system (Gemstar 6 XT, Arradiance) at 150 °C using TMA and H_2_O as precursors. Ultrahigh purity N_2_ was used as a carrier gas at a flow rate of 30 mL/min. In a typical synthesis, 100 mg of catalyst was placed in a sample tray degassed at 150 °C for 2 h before exposure to TMA and water vapors. The timing sequence was 30, 300, 30, and 300 s for TMA exposure, N_2_ purge, water exposure, and N_2_ purge, respectively.

All catalysts were calcined in air at 500 °C for 1 h (ramping rate of 10 °C/min) before characterizations and reaction tests.

### Catalytic Test

H/D exchange between H_2_ and D_2_ was conducted in a fixed-bed reactor system with 1/4 inch stainless steel reactor tube. Briefly, 10 mg of the catalyst was mixed with 200 mg of quartz sand, activated in 10% O_2_/Ar (100 mL/min) at 400 °C for 30 min, and then reduced in 10% H_2_/Ar (100 mL/min) at 200 °C for 30 min. After cooling down to 20 °C, 200 mL/min 5% H_2_/N_2_ and 200 mL/min 5% D_2_/N_2_ were fed into the reactor. Mass signals of *m*/*z* = 2, 3, 4 were monitored by SRS QMS200 mass spectrometer. The catalytic activity was calculated after 20 min of reaction.

Hydrogenation reactions were carried out in a fixed-bed reactor system with 1/4 inch quartz reactor tube. A thermocouple was inserted into the catalyst bed to control the reaction temperature.

For ethylene hydrogenation, 10 mg of the catalyst was mixed with 200 mg of quartz sand, activated in 10% O_2_/N_2_ (100 mL/min) at 400 °C for 30 min, and then reduced in 10% H_2_/N_2_ (100 mL/min) at 200 °C for 30 min. After cooling down to 20 °C, 37.5 mL/min 5% C_2_H_4_/N_2_ and 112.5 mL/min 5% H_2_/N_2_ were fed into the reactor. The reactor tube was flushed by cooling air to avoid temperature increase due to the exothermic effect of hydrogenation reactions. Agilent 490 microGC equipped with MS-5A (H_2_, O_2_, N_2_, CH_4_, CO), Plot U (CO_2_, C_2_H_2_, C_2_H_4_, and C_2_H_6_) and Alumina (C3+ alkanes and C3+ olefins) columns and three independent thermal conductivity detectors was used for online product analysis. N_2_ was used as the internal standard for GC quantification. The catalytic activity was calculated after 5 min of reaction.

For isobutene hydrogenation, 20 mg of the catalyst was mixed with 400 mg of quartz sand. The pretreatment and reaction conditions were the same with ethylene hydrogenation except that 25 mL/min 5% i-C_4_H_8_/N_2_ and 75 mL/min 5% H_2_/N_2_ were used as reactant gases.

### Characterizations

Aberration-corrected HAADF-STEM imaging was performed on a probe-corrected JEOL NEOARM operated at 80 kV. Dry powder specimens suspended on a Cu grid with a lacey carbon support were used for HAADF-STEM characterization. Scanning electron microscopy (SEM) was conducted on a Quanta 3D DualBeam FEG FIB-SEM with EDS analyzer to observe the zeolite morphology. Nitrogen sorption isotherms were measured at 77 K on a Micromeritics ASAP 2020 Plus (USA). The Brunauer–Emmett–Teller (BET) method was utilized to calculate the specific surface areas. Diffuse reflectance infrared Fourier transform spectroscopy (DRIFTS) measurements were performed on a Thermo Nicolet 6700 instrument with a Hg-Cd-Te (MCT) detector and a Praying Mantis high-temperature reaction chamber with KBr windows. The catalysts were pretreated with 100 mL/min of 10% O_2_/He at 400 °C for 30 min and then reduced by 10% H_2_/He at 200 °C for 30 min. The CO adsorption was performed at room temperature. Briefly, 5% CO/He was introduced into the DRIFTS cell at a flow rate of 100 mL/min. After the CO saturation, a helium purge at a flow rate of 100 mL/min was performed to remove gas-phase CO from the DRIFTS cell. All of the spectra were recorded using 32 scans and a resolution of 4 cm^–1^.

## Supporting Information Available

SEM images of all zeolites, detailed N_2_ sorption results, additional TEM and HAADF images of uncoated and coated Pt/zeolite samples, CO IR spectra, and catalysis results (PDF)
