# Supplementary material for: Construction of Inverse Metal–Zeolite Interfaces via Area-Selective Atomic Layer Deposition
Source: ACS Appl Mater Interfaces. 2021 Oct 20;13(43):51759–66. doi: 10.1021/acsami.1c15569 (PMC8569675; doi:10.1021/acsami.1c15569)
Supplement: Supplementary file 1 — am1c15569_si_001.pdf [file am1c15569_si_001.pdf]

Supporting Information

Construction of Inverse Metal-Zeolite Interfaces via Area-Selective Atomic Layer Deposition

Peng Zhai[1], Laibao Zhang[1], David A. Cullen[2], Divakar R. Aireddy[1], and Kunlun Ding[1]*

[1]Department of Chemical Engineering, Louisiana State University, Baton Rouge, LA 70803, USA.

[2]Center for Nanophase Materials Sciences, Oak Ridge National Laboratory, Oak Ridge, TN 37831, USA.

Correspondence to: kunlunding@lsu.edu

Contents:

SEM images of all zeolites

N_2 sorption results

TEM and HAADF images of uncoated and coated Pt/zeolite samples

CO IR spectra

Catalysis results

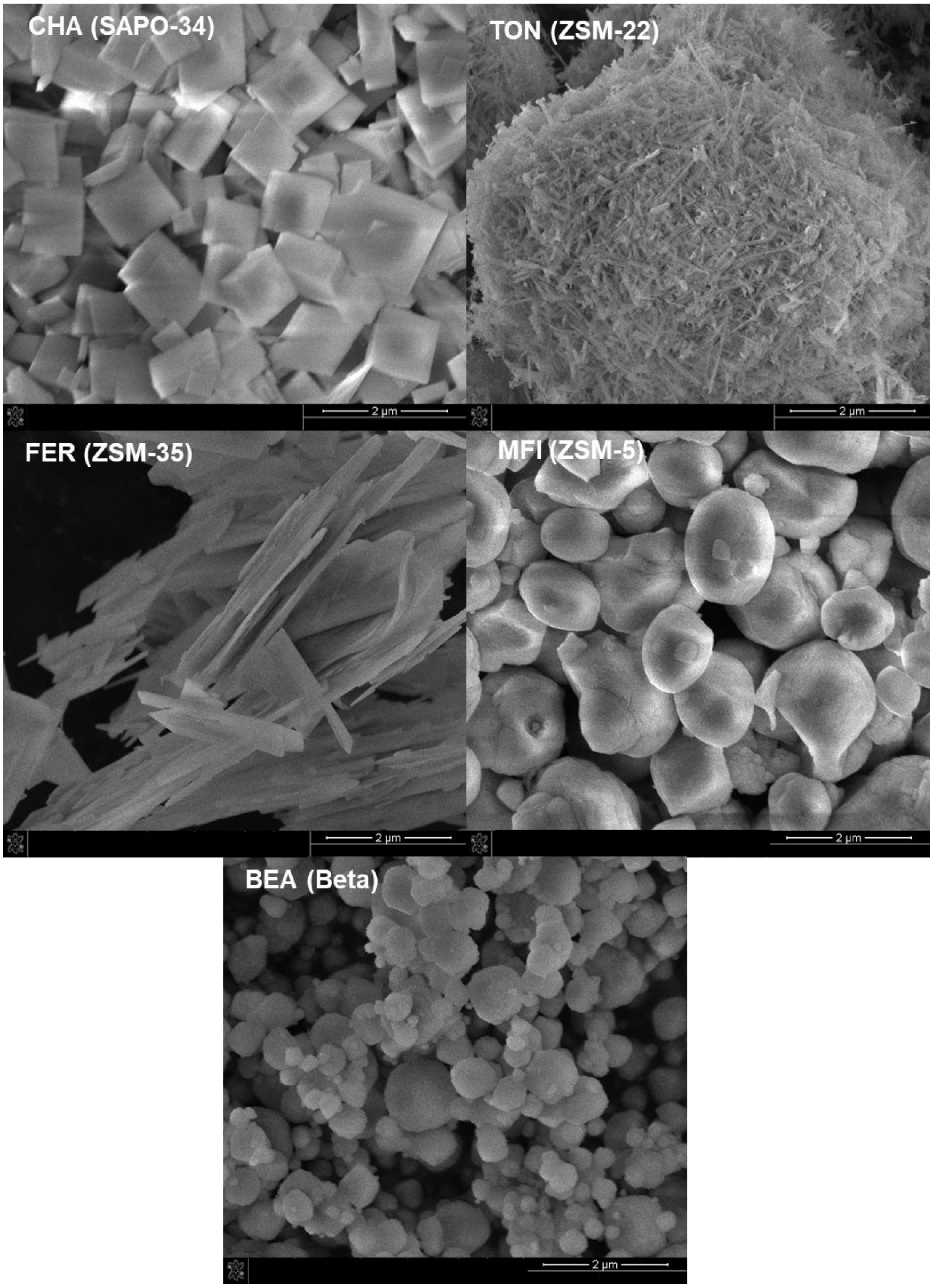

Figure S1. SEM images of all zeolites. All scale bars are 2 μm.

Table S1. Summary of nitrogen sorption results.

Sample names	Sample description	Single point pore volume (cm^3/g)	BET surface area (m^2/g)	t-plot micropore surface area (m^2/g)	t-plot external surface area (m^2/g)
SAPO-34	550°C calcined	0.40	531.8	476.9	55.0
SAPO-34	20-cycle AlO_x ALD, 500°C calcined	0.06	34.2	17.0	17.2
ZSM-22	550°C calcined	0.32	194.9	133.2	61.7
ZSM-22	20-cycle AlO_x ALD, 500°C calcined	0.14	31.3	4.8	26.5
ZSM-35	550°C calcined	0.08	253.5	216.7	36.8
ZSM-35	20-cycle AlO_x ALD, 500°C calcined	0.05	20.6	8.7	11.9
ZSM-5	550°C calcined	0.24	380.9	160.0	220.9
ZSM-5	20-cycle AlO_x ALD, 500°C calcined	0.02	32.2	18.3	13.9
Beta	550°C calcined	0.37	555.0	363.5	191.5
Beta	20-cycle AlO_x ALD, 500°C calcined	0.02	7.7	2.3	5.5
Pt/Beta	400°C calcined	0.36	546.5	361.4	185.1
Pt/Beta	20-cycle AlO_x ALD, 500°C calcined	0.03	15.9	4.2	11.6
Pt/Beta	Chlorosilane modification, 20-cycle AlO_x AS-ALD, 500°C calcined	0.22	339.7	260.3	79.4
Pt/Beta	Hexadecanol modification, 20-cycle AlO_x AS-ALD, 500°C calcined	0.16	235.2	173.3	61.8

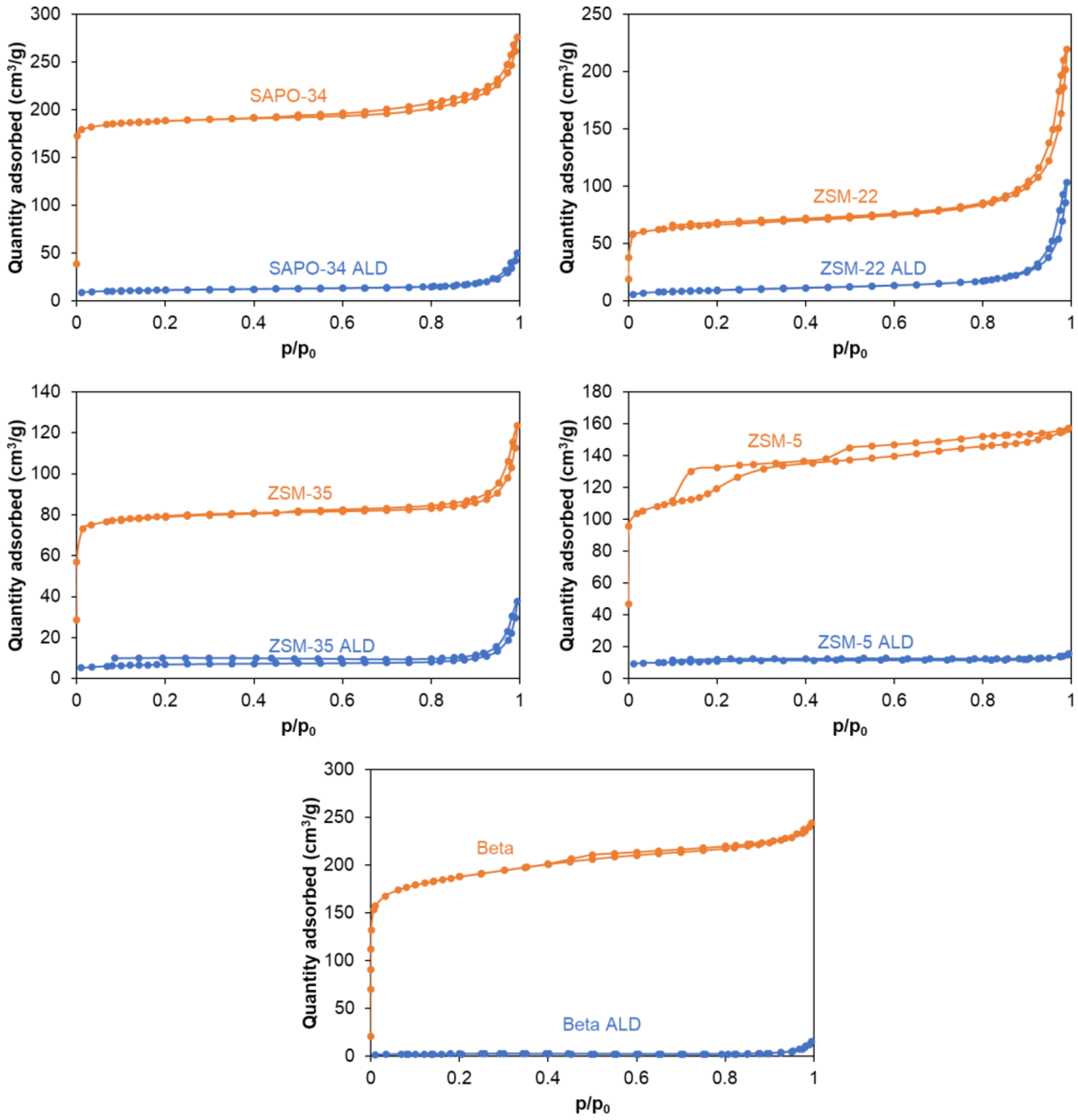

Figure S2. Nitrogen sorption isotherms of all zeolites before and after 20 cycles of AlO_x ALD. Uncoated zeolite samples were calcined at 550°C for 1 h. Zeolites after ALD were calcined at 500°C for 1 h.

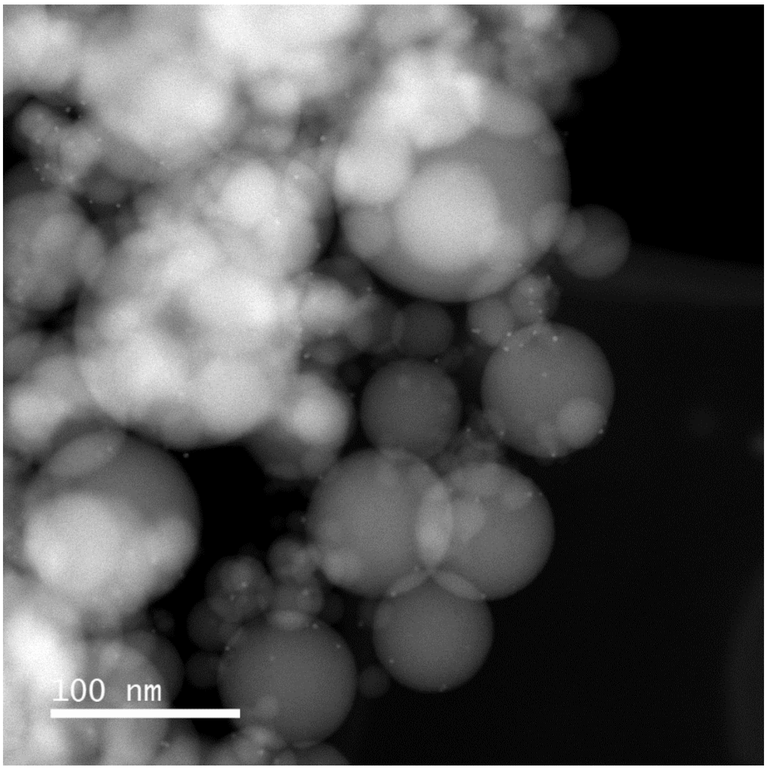

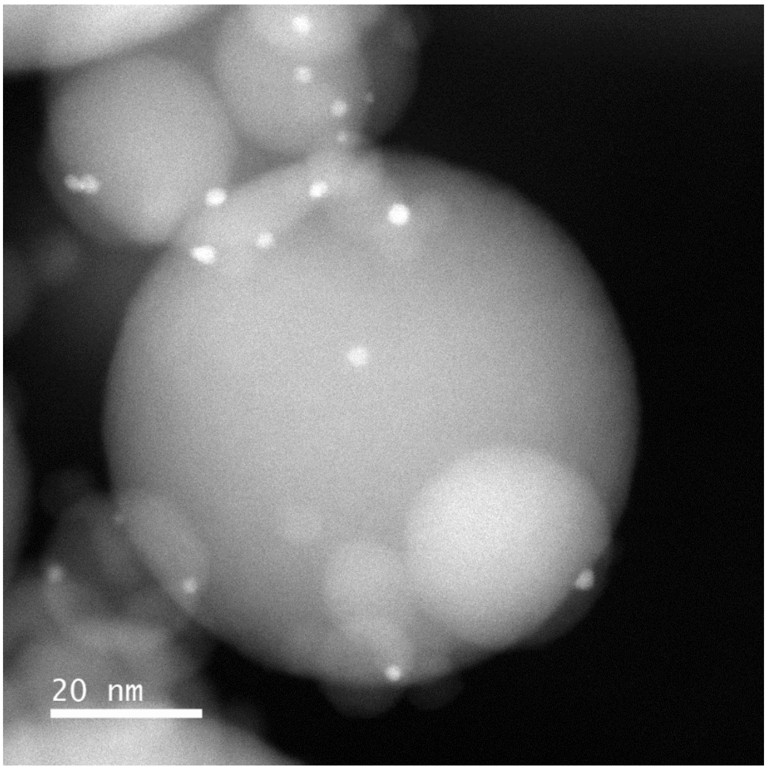

Figure S3. HAADF-STEM images of Pt/Al_2O_3 (calcined at 400°C).

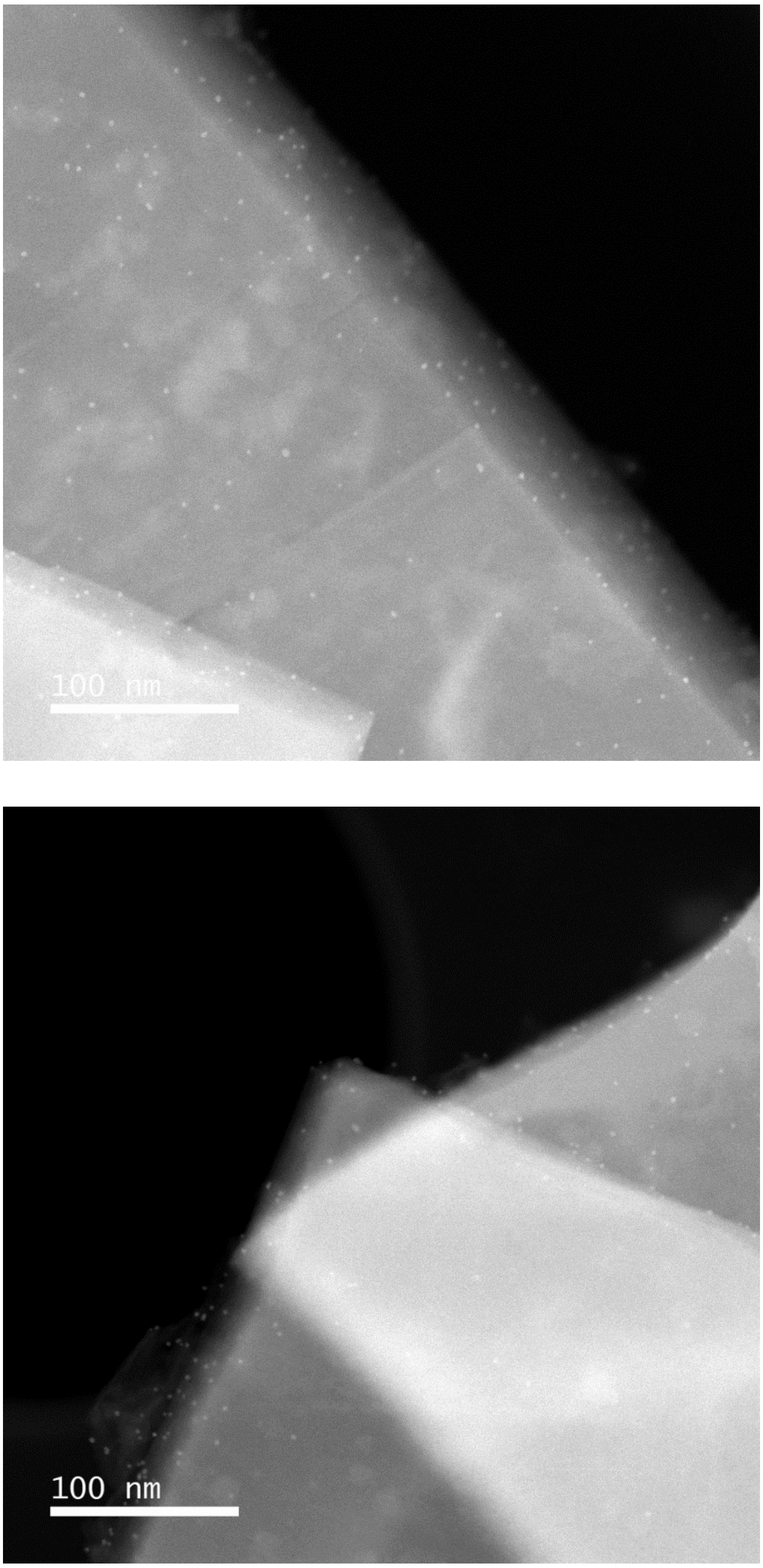

Figure S4. HAADF-STEM images of Pt/CHA (SAPO-34, calcined at 400℃).

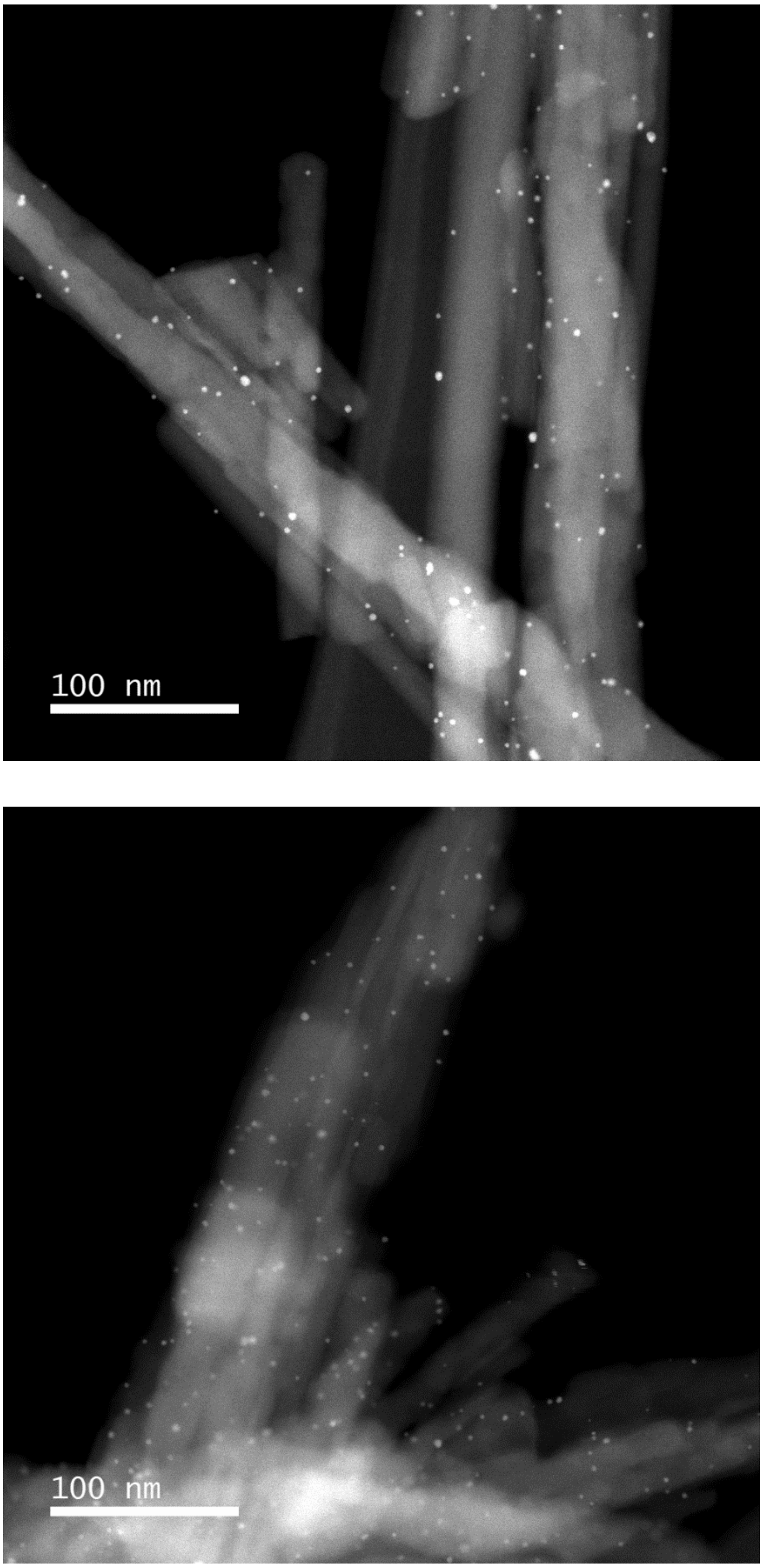

Figure S5. HAADF-STEM images of Pt/TON (ZSM-22, calcined at 400°C).

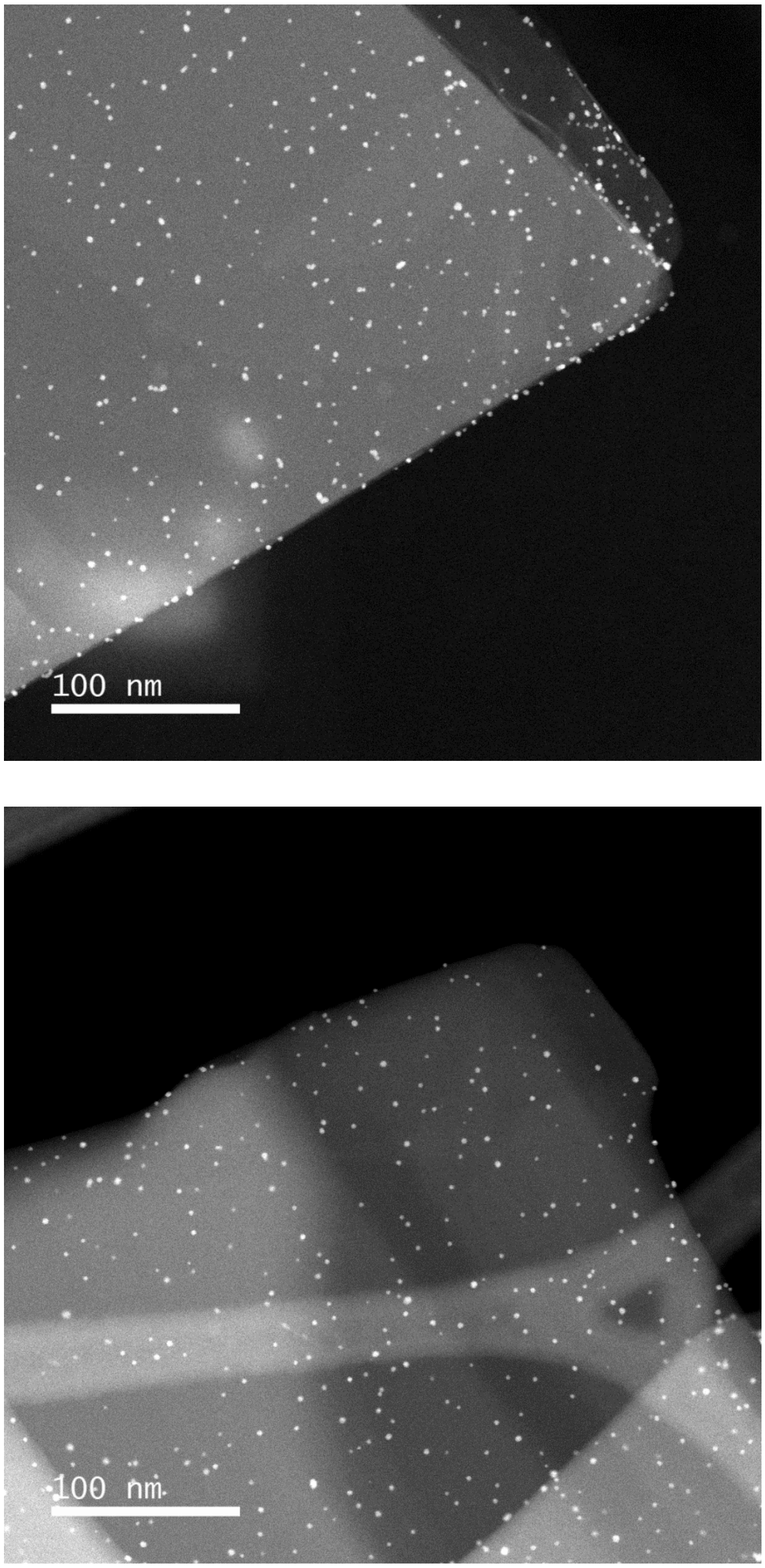

Figure S6. HAADF-STEM images of Pt/FER (ZSM-35, calcined at 400℃).

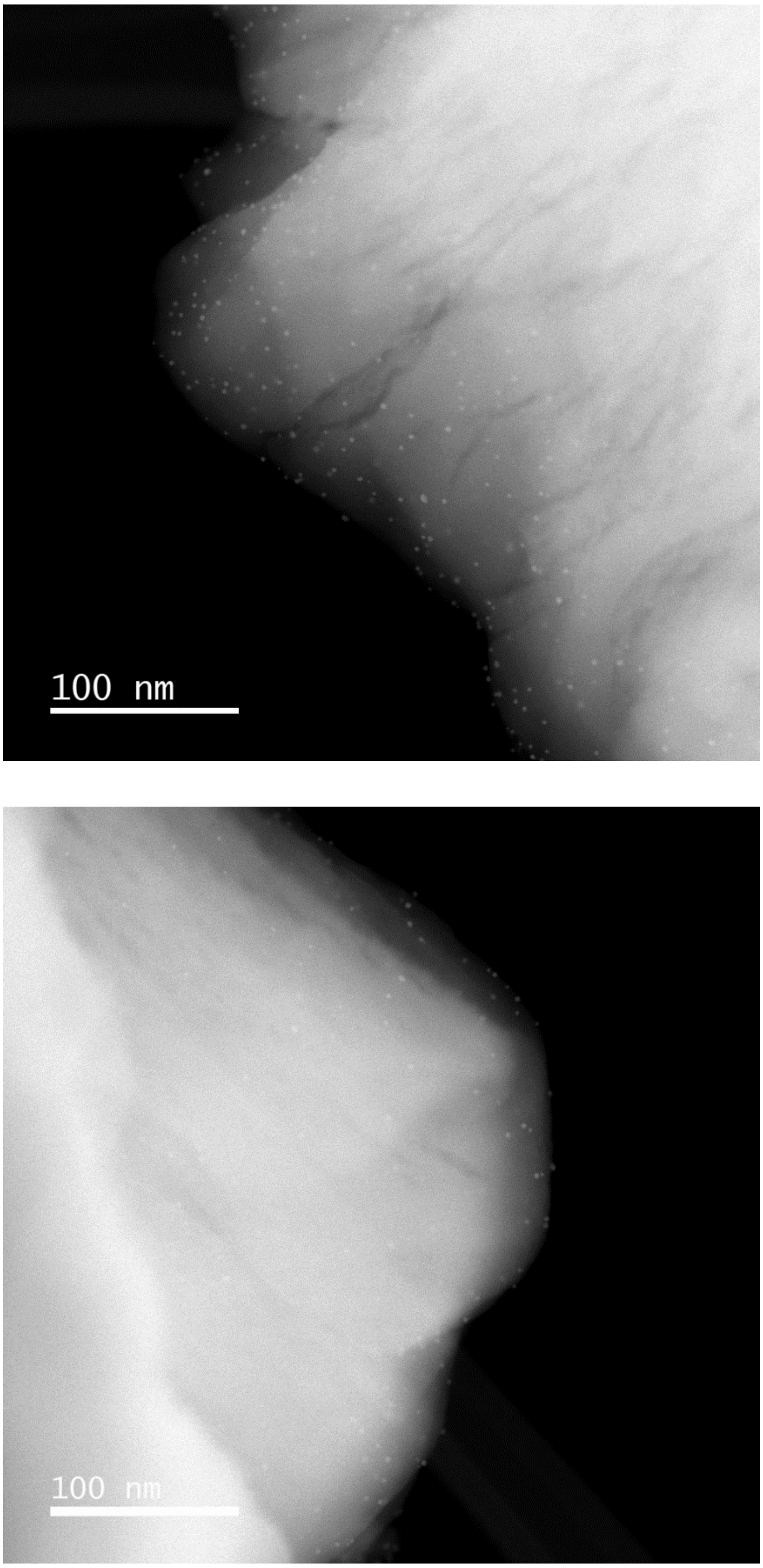

Figure S7. HAADF-STEM images of Pt/MFI (ZSM-5, calcined at 400°C).

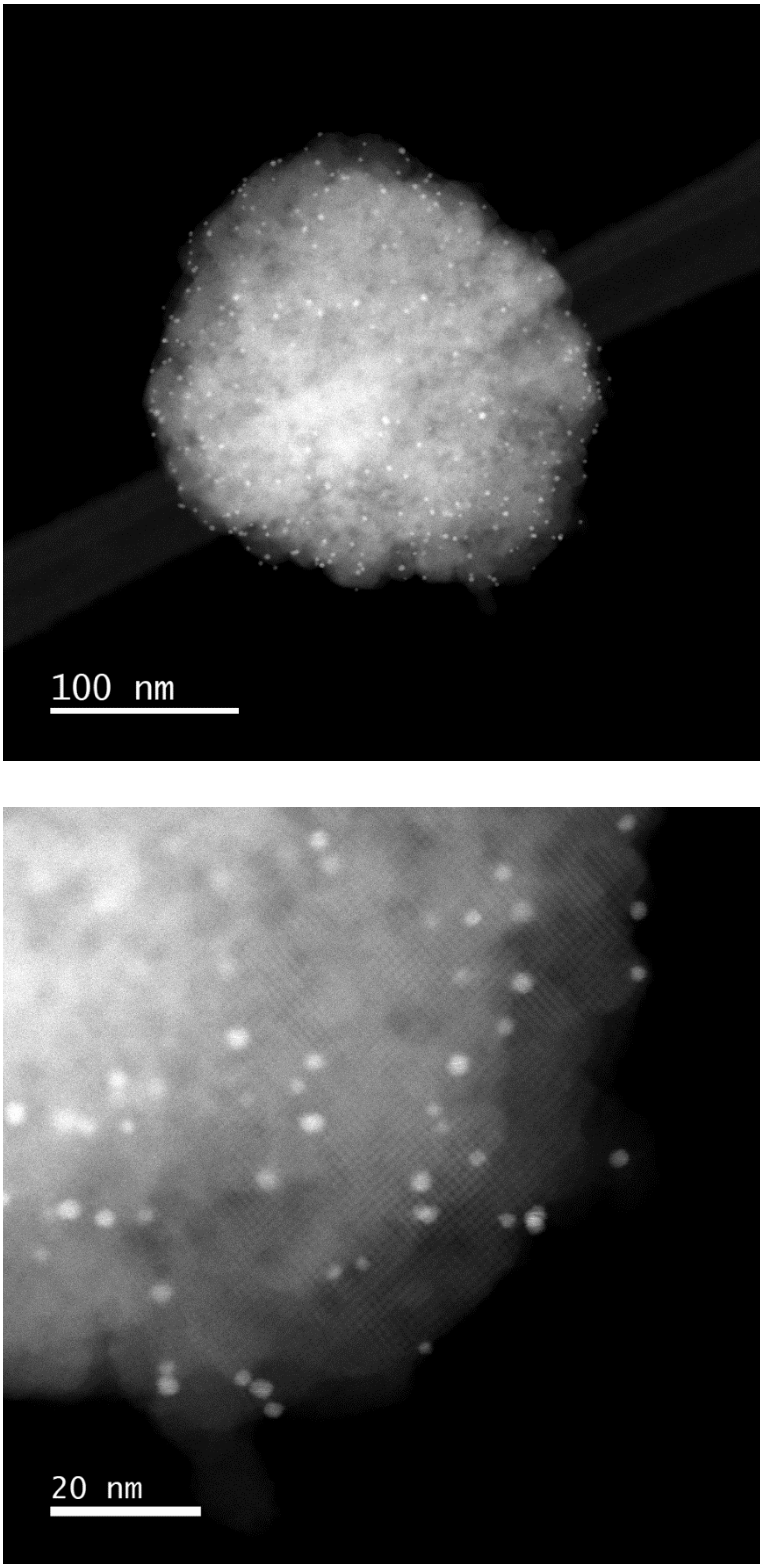

Figure S8. HAADF-STEM images of Pt/BEA (Beta, calcined at 400°C).

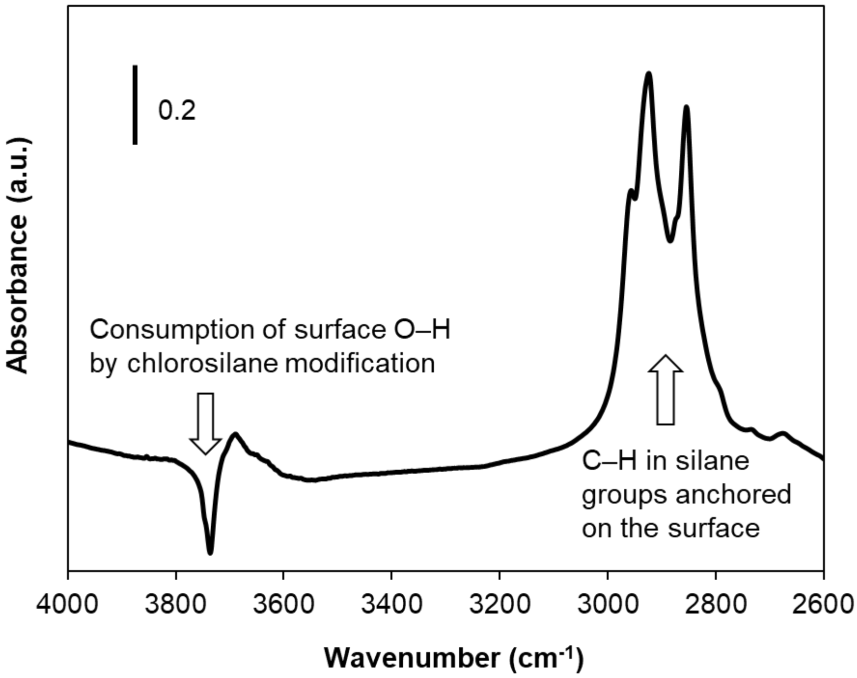

Figure S9. IR spectrum of chlorosilane-modified Pt/BEA using calcined Pt/BEA as background.

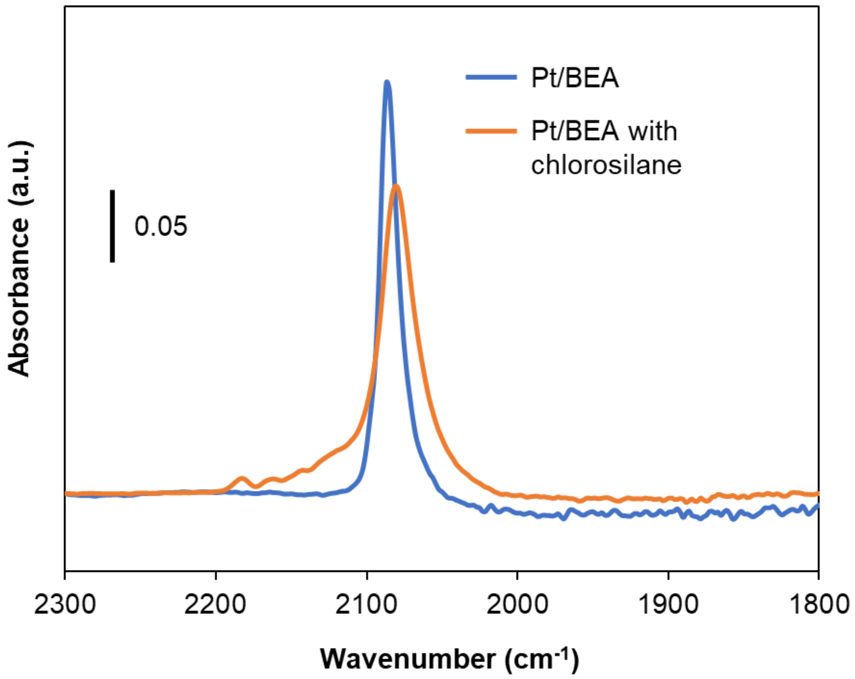

Figure S10. IR spectra of CO molecules adsorbed on Pt/BEA with and without chlorosilane modification. Note that the CO band became slightly broader with no obvious change in peak area upon chlorosilane modification.

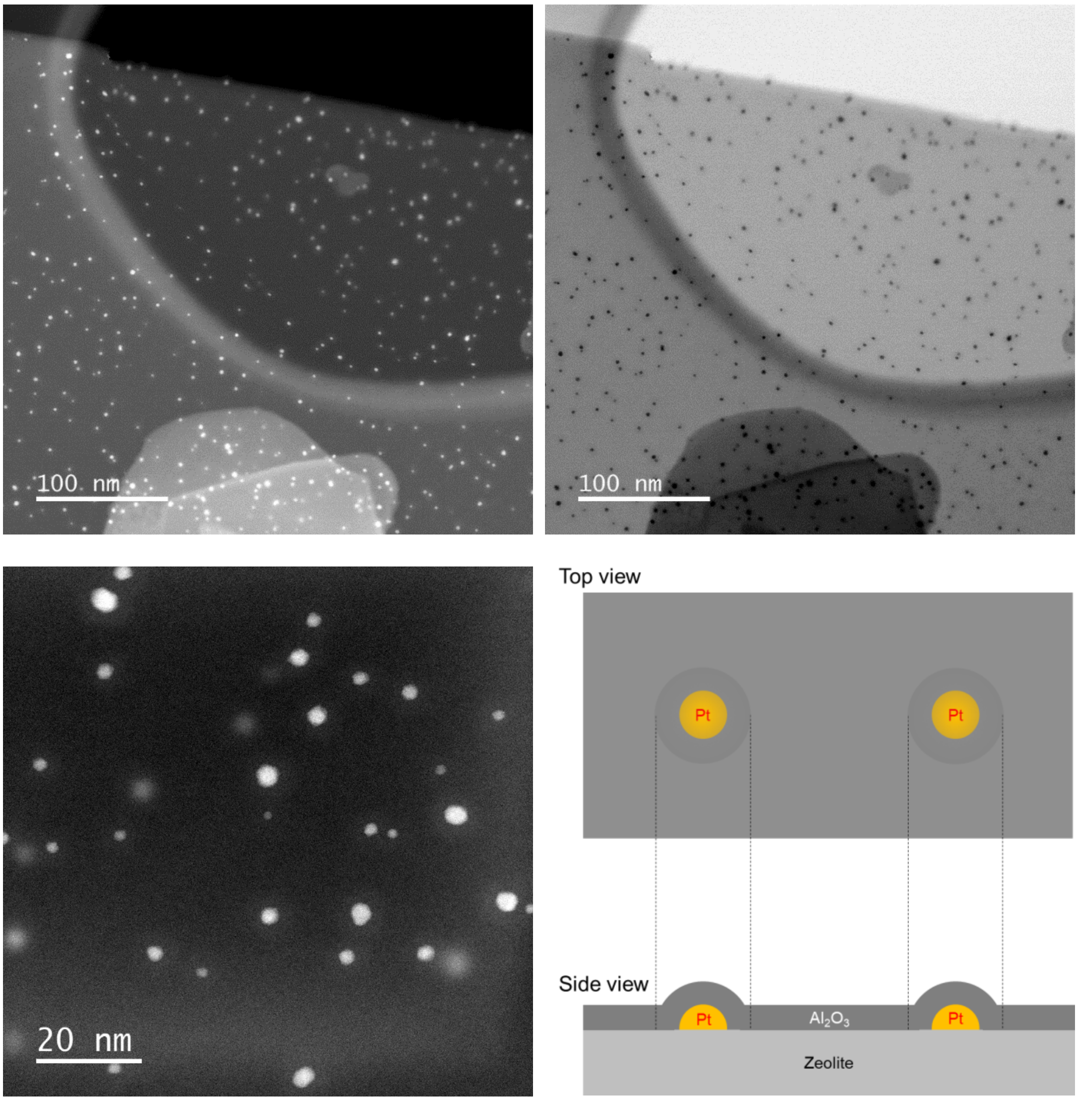

Figure S11. HAADF-STEM (top-left and bottom-left) and bright-field STEM (top-right) images of Pt/ZSM-35 after ALD (without using blocking agents, calcined at 500°C). The light halo structures around Pt NPs are barely noticeable in these images. Bottom-right illustrates the origin of light halo structures.

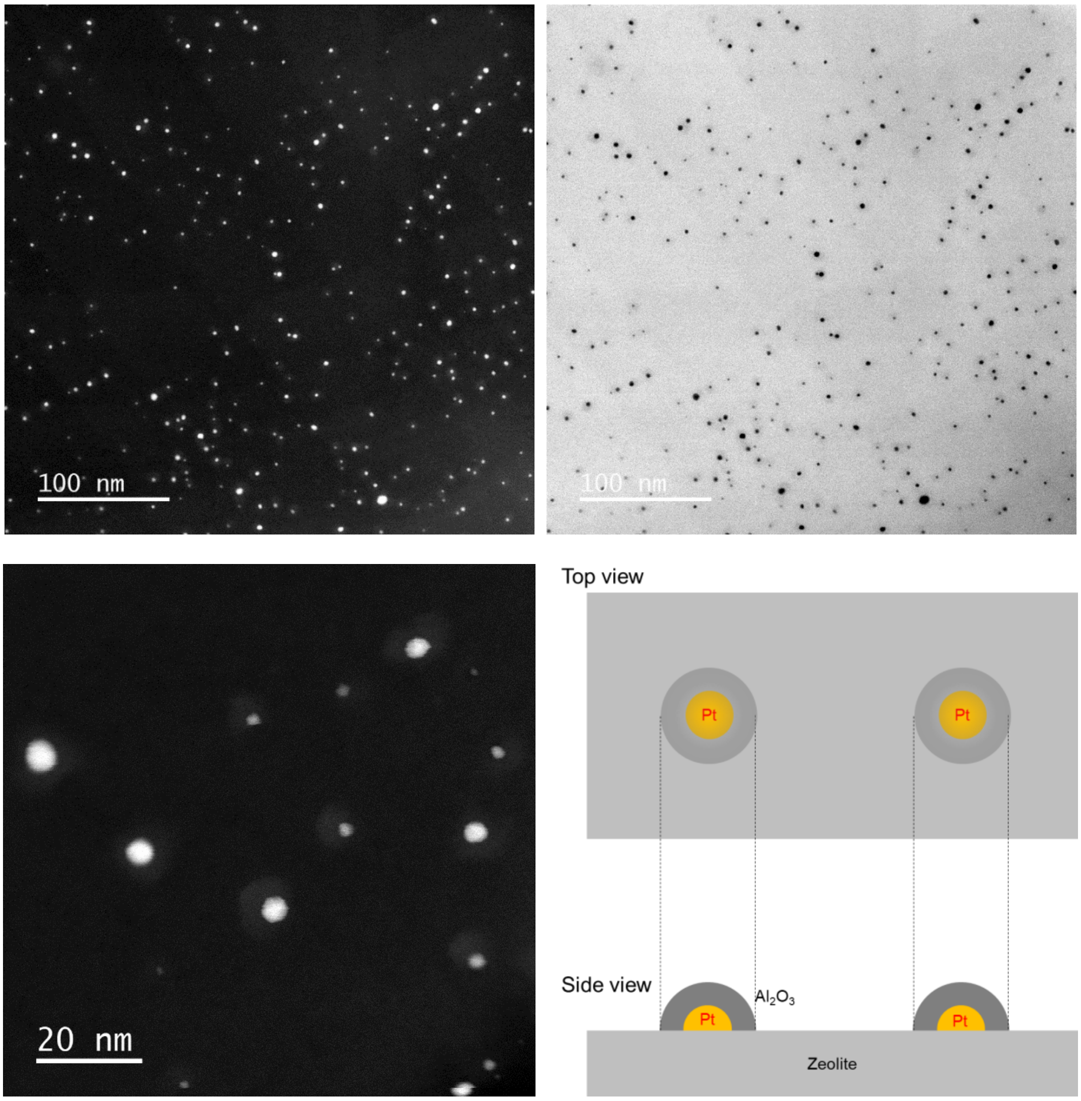

Figure S12. HAADF-STEM (top-left and bottom-left) and bright-field STEM (top-right) images of Pt/ZSM-35 after AS-ALD (using chlorosilane as a blocking agent, calcined at 500°C). Halo structures around Pt NPs can be observed. Bottom-right illustrates the origin of halo structures.

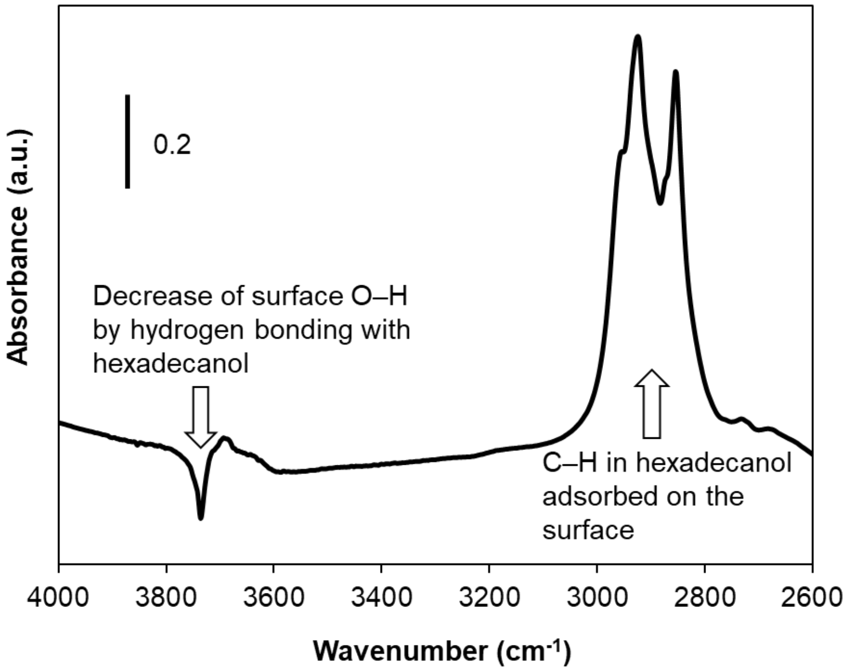

Figure S13. IR spectrum of hexadecanol-modified Pt/BEA using calcined Pt/BEA as background.

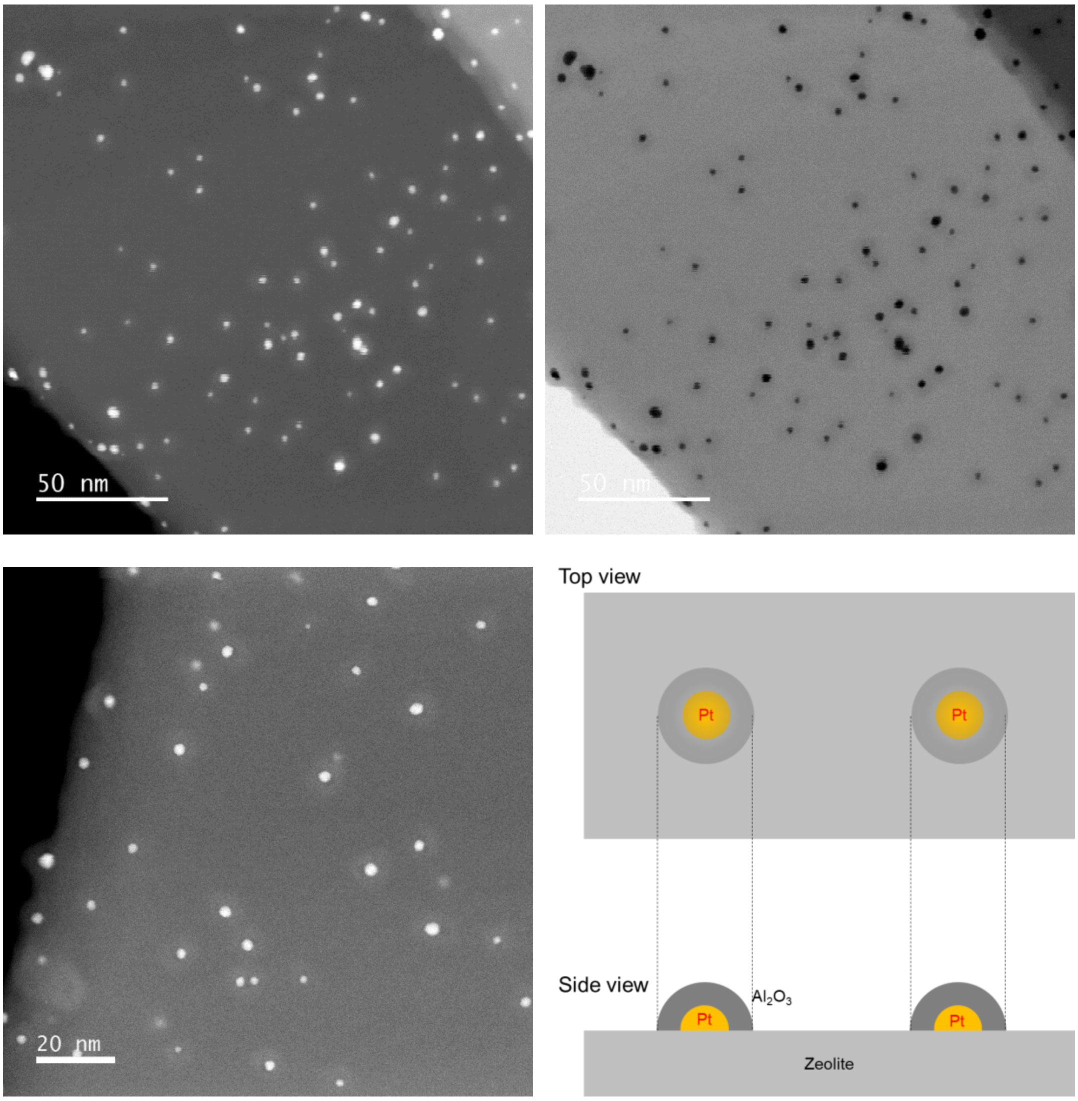

Figure S14. HAADF-STEM (top-left and bottom-left) and bright-field STEM (top-right) images of Pt/ZSM-35 after AS-ALD (using hexadecanol as a blocking agent, calcined at 500°C). Halo structures around Pt NPs can be observed. Bottom-right illustrates the origin of halo structures.

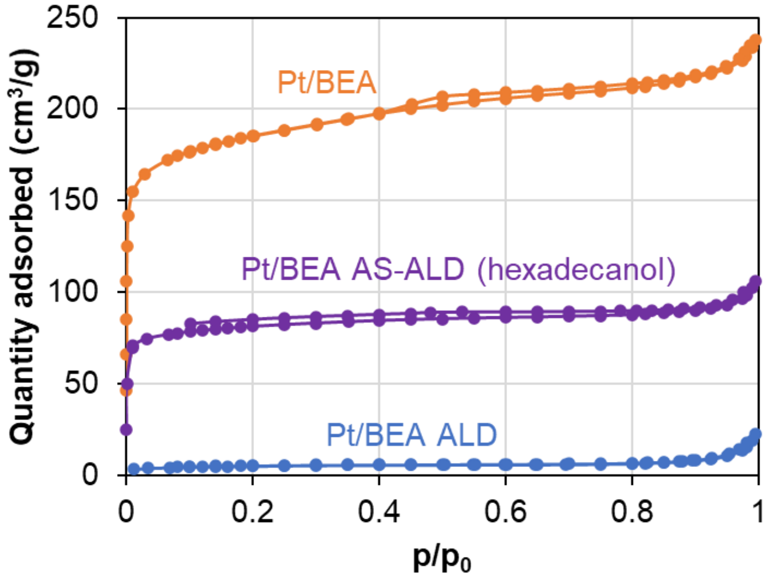

Figure S15. Nitrogen sorption isotherms of Pt/BEA, Pt/BEA after 20-cycle of AlO_x ALD, and Pt/BEA after 20-cycle of AlO_x AS-ALD using hexadecanol as a blocking agent. Pt/BEA was calcined at 400°C for 1 h. The other two samples were calcined at 500°C for 1 h.

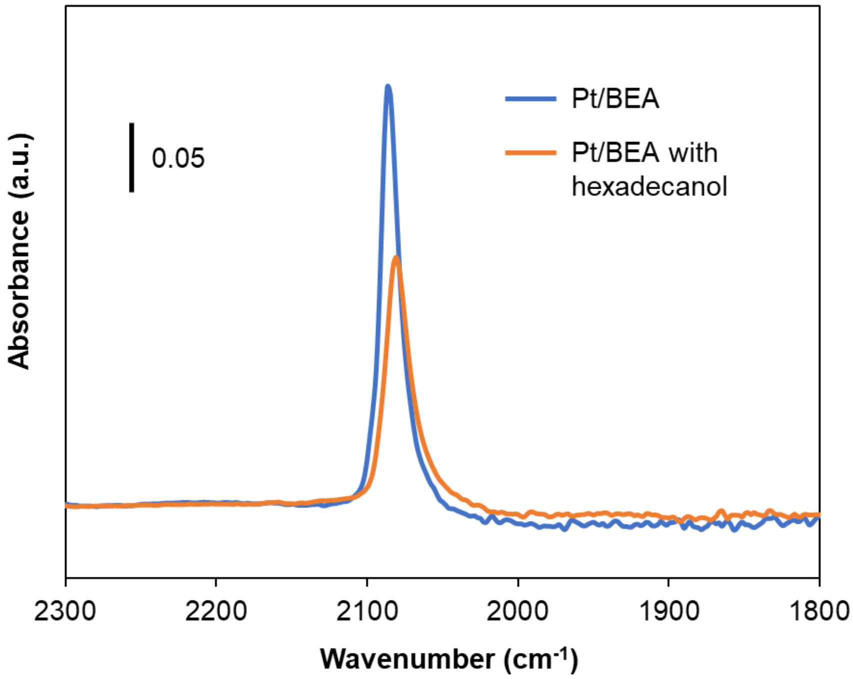

Figure S16. IR spectra of CO molecules adsorbed on Pt/BEA with and without hexadecanol modification. Note that the CO band area dropped by 20-30% upon hexadecanol modification.

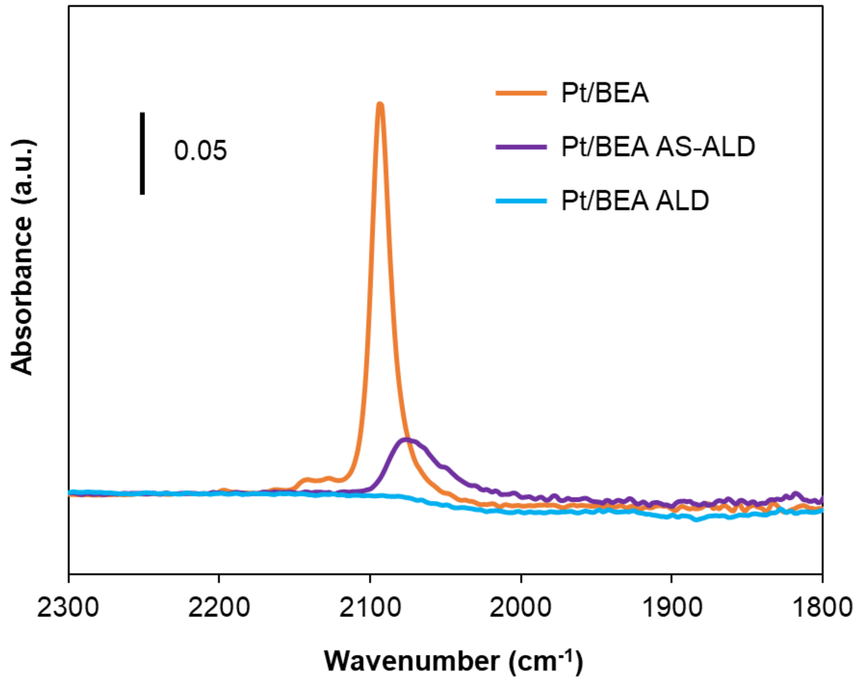

Figure S17. IR spectra of CO molecule adsorbed on Pt/BEA and that after 20-cycle of AlO_x ALD or AS-ALD using hexadecanol as a blocking agent. Pt/BEA was calcined at 400°C for 1 h and reduced in hydrogen at 200°C for 30 min prior to CO adsorption. The other two samples were calcined at 500°C for 1 h and then reduced in hydrogen at 200°C for 30 min prior to CO adsorption.

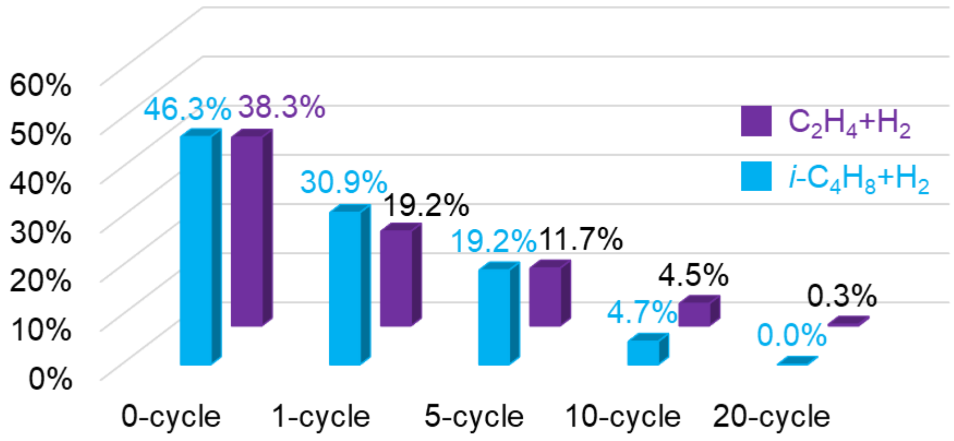

Figure S18. Catalytic performance of Pt/Al_2O_3 in ethylene and isobutene hydrogenation after different numbers of cycles of AlO_x ALD. 20 cycles are needed to completely encapsulate Pt NPs.

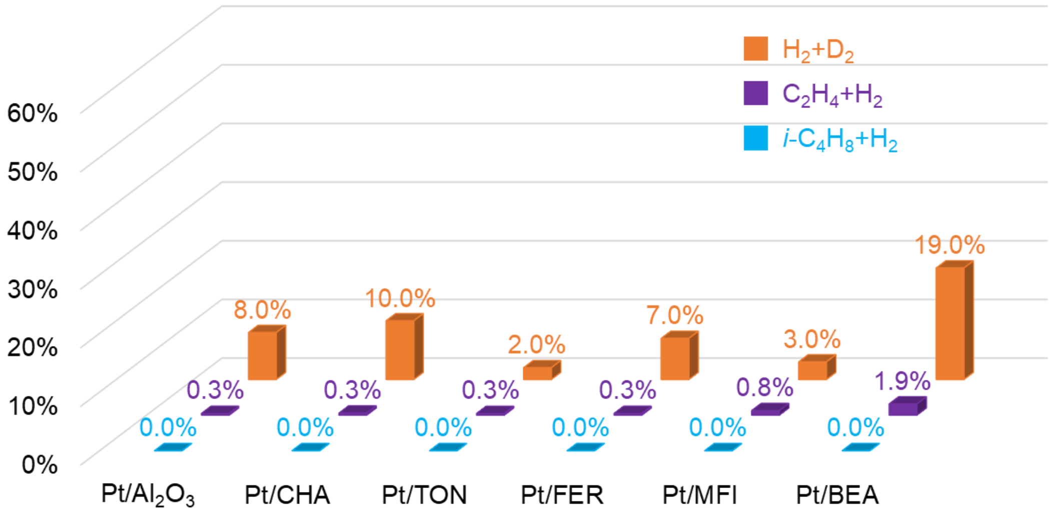

Figure S19. Catalytic performance of different catalysts after 20 cycles of AlO_x ALD without using blocking agents.

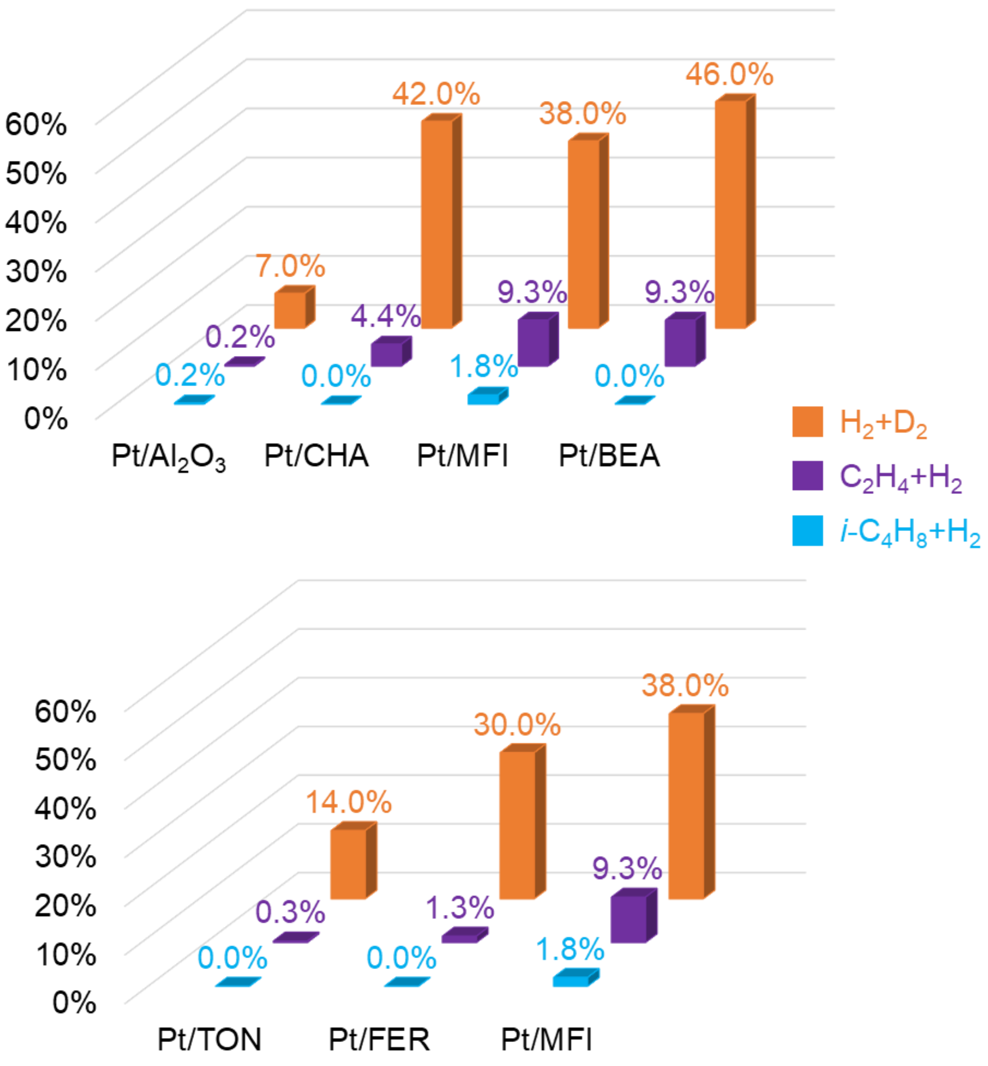

Figure S20. Catalytic performance of different catalysts after 20 cycles of AlO_x AS-ALD using hexadecanol as a blocking agent.
